# Use of a Real-Time Locating System for Contact Tracing of Health Care Workers During the COVID-19 Pandemic at an Infectious Disease Center in Singapore: Validation Study

**DOI:** 10.2196/19437

**Published:** 2020-05-26

**Authors:** Hanley J Ho, Zoe Xiaozhu Zhang, Zhilian Huang, Aung Hein Aung, Wei-Yen Lim, Angela Chow

**Affiliations:** 1 Department of Clinical Epidemiology Office of Clinical Epidemiology, Analytics, and Knowledge (OCEAN) Tan Tock Seng Hospital Singapore; 2 Lee Kong Chian School of Medicine Nanyang Technological University Singapore

**Keywords:** infectious disease, real-time locating systems, electronic medical records, COVID-19, contact tracing, health care workers, RFID

## Abstract

**Background:**

In early 2020, coronavirus disease (COVID-19) emerged and spread by community and nosocomial transmission. Effective contact tracing of potentially exposed health care workers is crucial for the prevention and control of infectious disease outbreaks in the health care setting.

**Objective:**

This study aimed to evaluate the comparative effectiveness of contact tracing during the COVID-19 pandemic through the real-time locating system (RTLS) and review of the electronic medical record (EMR) at the designated hospital for COVID-19 response in Singapore.

**Methods:**

Over a 2-day study period, all admitted patients with COVID-19, their ward locations, and the health care workers rostered to each ward were identified to determine the total number of potential contacts between patients with COVID-19 and health care workers. The numbers of staff-patient contacts determined by EMR reviews, RTLS-based contact tracing, and a combination of both methods were evaluated. The use of EMR-based and RTLS-based contact tracing methods was further validated by comparing their sensitivity and specificity against self-reported staff-patient contacts by health care workers.

**Results:**

Of 796 potential staff-patient contacts (between 17 patients and 162 staff members), 104 (13.1%) were identified by both the RTLS and EMR, 54 (6.8%) by the RTLS alone, and 99 (12.4%) by the EMR alone; 539 (67.7%) were not identified through either method. Compared to self-reported contacts, EMR reviews had a sensitivity of 47.2% and a specificity of 77.9%, while the RTLS had a sensitivity of 72.2% and a specificity of 87.7%. The highest sensitivity was obtained by including all contacts identified by either the RTLS or the EMR (sensitivity 77.8%, specificity 73.4%).

**Conclusions:**

RTLS-based contact tracing showed higher sensitivity and specificity than EMR review. Integration of both methods provided the best performance for rapid contact tracing, although technical adjustments to the RTLS and increasing user compliance with wearing of RTLS tags remain necessary.

## Introduction

In early 2020, coronavirus disease (COVID-19) emerged in Wuhan, China; the disease spread by community and nosocomial transmission, infecting up to 3019 health care workers by February 12, 2020 [1].

Contact tracing is used to identify individuals potentially exposed to infectious diseases; it is crucial for the prevention and control of infectious disease outbreaks [2,3]. Health care workers face high risks of contracting infectious diseases due to the large amounts of time they spend interacting with patients and their coworkers [4,5]. Health care workers experience contact with 14-18 persons in a typical work shift in a general ward, with nurses having the longest duration of physical contact with patients [6]. As contacts in health care settings tend to be close, any exposure to an infectious patient requires immediate contact tracing and contact management. Failure to identify potentially exposed contacts puts other patients and health care workers at greater risk of infection [7].

Conventional contact tracing methods are limited by their cost and reliability. Continuous direct observation has been considered to be the gold standard method to accurately quantify contact time (including activities and locations of interest); however, due to the intensive human resource requirements of this method, it is cost-ineffective and impractical for large-scale projects [8]. Self-reporting methods, such as activity diaries and interviews, have been used as alternatives to direct observation due to the lower intensity of their human resource demands [6,8]; however, these methods are also time-consuming and subject to reporting biases that compromise the accuracy of the data collected [9,10]. Another commonly used method for contact tracing is data extraction from administrative and clinical databases such as electronic medical records (EMRs) [8,11]. Although this method overcomes some of the problems related to cost and reliability, it is also time-consuming and limits the capture of patient contact episodes to care providers who can input data in the EMR [8,12].

Technological advances such as real-time locating systems (RTLSs) have shown promise in overcoming the barriers to conventional contact tracing methods [13,14]. In recent years, health care institutions have been increasingly exploring the use of RTLSs to establish contacts within health care premises [12,13,15]. One type of RTLS technology is radio-frequency identification (RFID) tracking. RFID tracking requires the user to wear an RFID tag, which continuously sends wireless signals to sensors (RFID readers) installed at various locations in the hospital [16]. RFID technology can provide an accurate gauge of health care workers’ movements and their interactions with patients and coworkers [15]. This capability is useful for hospital contact tracing during an infectious disease outbreak.

While many studies have been conducted on community-based contact tracing measures using digital technology, fewer articles have focused on technology for hospital-based contact tracing for health care workers. During the COVID-19 pandemic, we evaluated the comparative effectiveness of contact tracing through RTLSs and the conventional method of identifying contacts from EMRs and validated these methods against self-reporting of contacts by health care workers.

## Methods

### Setting

This study was performed during the COVID-19 pandemic in Singapore at the National Centre for Infectious Diseases (NCID), the designated hospital for COVID-19 response. Singapore identified its first COVID-19 case on January 23, 2020; by February 19, 2020, a total of 84 cases were reported nationally [17]. Of these, the NCID managed 65 cases. The study was conducted over 2 days from midnight on February 25, 2020 to 11:59 PM on February 26, 2020. The NCID is a 330-bed purpose-built facility for the management of emerging infectious diseases. The NCID building was outfitted with RTLS technology, and location trackers were installed in all ward areas. All staff working within the building were provided with RTLS tags, which they routinely carried during work. The tags also served as access cards to the NCID building and inpatient wards.

### Real-Time Locating System

Staff wearing RTLS tags could be located within the wards and inpatient rooms of the NCID. All inpatient rooms were fitted with RTLS location exciters and wireless access points. Whenever a tag passed a location exciter in an inpatient room, the tag would receive a low-frequency signal and transmit a radio frequency signal to the access point, where the location triangulation technology would decipher the signals and determine the exact location of the RTLS tag.

### Electronic Medical Record

The NCID also uses an EMR system to capture clinical encounters of all inpatients. Staff providing clinical care were issued with personal accounts to make entries into patients’ clinical notes regarding their clinical assessments, medication orders, laboratory and radiological tests, and charting of vital signs.

### Participants

Participants included medical and nursing staff who could have had contact with patients with COVID-19 isolated in the general wards of the NCID during the 2-day study period. Inpatients with a laboratory-confirmed diagnosis of COVID-19 infection were identified through the hospital’s laboratory information system, and on-duty medical and nursing staff were identified from duty rosters.

We excluded staff members who did not have access to the hospital EMR system, did not have a working RTLS tag associated with their identity, or did not have a sufficiently charged RTLS tag that could be reliably detected by the location trackers. We identified these staff members by checking all staff that were on the roster for any movement records captured by the RTLS in the week preceding the 2 days of the study, and we excluded those whose identity could not be found in the RTLS (ie, they were not issued a tag) or who did not register any movement record (ie, they were not using their tag or their tag had not been charged).

### Study Design

The study was divided into two parts. In the first part of the study, we compared two methods of contact tracing for medical and nursing staff who had come into contact with a confirmed patient with COVID-19 over the study period: the conventional method of reviewing the EMR and the extraction of staff records from the RTLS. For the EMR reviews, we extracted the names of all physicians and nurses who had come into contact with patients based on entries in the EMR. Records included medical and nursing notes as well as documentation of patients’ vital signs. For extraction of staff records from the RTLS, we used location-based tracking to identify all individuals picked up by the appropriate location tracker in a specific airborne infection isolation room to which each patient with COVID-19 was admitted. We used a highly sensitive cutoff of at least 1 second (ie, as long as a staff member’s RTLS tag was picked up by the location tracker in a specific room, we considered that the staff member had been in contact with the patient admitted to that room).

For each patient day (a 24-hour period from midnight to 11:59 PM for each specific patient with COVID-19), we counted the total number of unique staff-patient contacts identified using EMR-based and RTLS-based contact tracing methods. For staff with multiple contacts with the same patient in the same day, we included only the first contact episode. We then classified the episodes into contacts identified through both methods and contacts identified through either one of the two methods. We constructed 2×2 tables for each patient day and aggregated these tables. We reported the total number of contacts identified by the two methods, the proportions identified by both methods, and the proportions identified by either one of the two methods.

In the second part of our study, we attempted to validate both methods against self-reporting by physicians and nurses. For this part of the study, we restricted our analysis only to patients with COVID-19 who had been admitted to two wards in the NCID on February 25, 2020. We identified all possible contacts of a patient by examining the rostered medical and nursing staff for a given ward. We then separately used both the EMR review and RTLS staff record extraction methods as detailed above to identify all medical and nursing staff members who had come into contact with each patient with COVID-19. For the self-reporting method, we contacted all physicians and nursing staff members rostered to those wards by telephone and asked them if they had physically entered the airborne infection isolation room (not just the anteroom) of each patient with COVID-19. This question was asked on the day after the day of interest to reduce recall bias. Our hospital protocol requires all staff managing patients with COVID-19 to don full personal protection equipment (including a fit-tested N95 mask, gown, gloves, and goggles/face shield) during their encounters with patients. This requirement is strictly enforced in the wards by both the ward nursing manager and senior physicians. As such, these staff members were not in danger of being quarantined or of being reprimanded for not following protocol. We therefore expected these self-reports to be truthful.

For each patient day, we constructed two 2×2 tables: one comparing the performance of the EMR review method against self-reported contacts (ie, its comparative ability to correctly identify staff-patient contacts and non–staff-patient contacts), and the other comparing the RTLS method against self-reported contacts. Tables for all patient days were then aggregated. We used the aggregated tables to calculate the sensitivity (proportion of correctly identified staff-patient contacts) and specificity (proportion of correctly identified non–staff-patient contacts) of both methods separately. In addition, we created three logistic regression models in which the dependent variable was the self-reported contacts: in Model 1, the only independent variable was EMR detection, in Model 2, the only independent variable was RTLS detection, and in Model 3, both variables were included as independent variables. We then used the likelihood ratio test to compare the goodness of fit of these models.

We further considered the sensitivity and specificity of using both methods concurrently, using either an “RTLS or EMR” approach or an “RTLS and EMR” approach, to detect staff-patient contacts.

## Results

### RTLS Versus EMR-Based Contact Tracing

Our study included 17 inpatients with COVID-19 warded at the NCID on February 25 and 26, 2020, housed in single airborne infection isolation rooms across six isolation wards. From the ward duty rosters, a total of 212 staff members (30 medical, 14.2%, and 182 nursing, 85.8%) were rostered for duty in these six wards over the study period. We excluded 50/212 staff members (23.6%; 5 medical, 10.0%, and 45 nursing, 90.0%) due to tag-related issues, and the remaining 162 staff members (25 medical, 15.4%, and 137 nursing, 84.6%) were included in our study. In total, based on the ward location of each patient and the number of staff rostered to each ward over the 2-day study period, 796 potential staff-patient contacts were identified between these 17 inpatients and 162 staff members.

Table 1 compares the number of contacts identified by the RTLS with those identified by the EMR for 34 patient days. Of 796 potential staff-patient contacts, 104 (13.1%) were identified by both RTLS and EMR, 54 (6.8%) by RTLS alone, and 99 (12.4%) by EMR alone; 539 (67.7%) were not identified through either method. Among the total of 257 staff-patient contacts identified, the RTLS identified 158 (61.5%), while the EMR identified 203 (79.0%). However, it is not possible to determine whether the contacts were identified accurately.

**Table 1:** Summary of possible contacts identified by the RTLS and EMR over 34 patient days. Percentages are calculated according to row values.

EMR detection status	Detected by RTLS^a^	Not detected by RTLS	Total
Detected by EMR^b^	104 (13.1%)	99 (12.4%)	203
Not detected by EMR	54 (6.8%)	539 (67.7%)	593
Total	158	638	796

^a^RTLS: real-time locating system.

^b^EMR: electronic medical record.

### Validation of RTLS-Based and EMR-Based Contact Tracing

For our validation study, we evaluated staff-patient contacts for 10 confirmed patients with COVID-19 in two wards (5 in Ward A and 5 in Ward B). During the 1-day validation study period, 36 staff members (6 medical, 17%, and 30 nursing, 83%) were rostered to Ward A and 30 staff members (6 medical, 20%, and 24 nursing, 80%) were rostered to Ward B. Of these, 8 staff members (1 medical, 13%, and 7 nursing, 87%) from Ward A and 2 staff members (both nursing) from Ward B were excluded due to staff tag–related issues. Therefore, we included 28 staff members from Ward A and 28 staff members from Ward B in our study. This gave a total of 280 potential staff-patient contacts.

In general, EMR review produced more staff records than the RTLS (*P*<.001). The overall observed agreement between the RTLS and EMR was 80.8%.

Table 2 compares the performance of RTLS staff records with self-reported contacts. Of 280 potential staff-patient contacts, 36 (12.9%) were self-reported as having occurred. Of these, 26 contacts were traced by RTLS, giving a sensitivity of 72.2%. Of 244 self-reported non–staff-patient contacts, 214 were accurately identified by RTLS, giving a specificity of 87.7%. The positive predictive value was 46.4%, while the negative predictive value was 95.5%.

Table 3 compares the performance of EMR review against self-reporting by staff. Of 36 self-reported staff-patient contacts, 17 were identified by EMR review, giving a sensitivity of 47.2%. Of 244 staff-patient contacts not reported by staff, 190 were identified by EMR review, giving a specificity of 77.9%. The positive predictive value was 23.9%, while the negative predictive value was 90.9%.

**Table 2:** Comparison of the performance of RTLS-based contact tracing with self-reported contacts with patients with COVID-19. Percentages are calculated according to row values.

Self-reported status	Detected by RTLS^a^	Not detected by RTLS	Total
Contacts by self-report	26 (72.2%)	10 (27.8%)	36
Noncontacts by self-report	30 (12.3%)	214 (87.7%)	244
Total	56	224	280

^a^RTLS: real-time locating system.

**Table 3:** Comparison of the performance of EMR-based contact tracing against self-reported contacts with patients with COVID-19. Percentages are calculated according to row values.

Self-reported status	Detected by EMR^a^	Not detected by EMR	Total
Contacts by self-report	17 (47.2%)	19 (52.8%)	36
Noncontacts by self-report	54 (22.1%)	190 (77.9%)	244
Total	71	209	280

^a^EMR: electronic medical record.

Among the logistic regression models, Model 3 performed significantly better than Model 1 (*P*<.001) but not differently from Model 2 (*P*=0.84) (Table 4). These results suggest that the RTLS was better than the EMR at identifying self-reported contacts. This is consistent with our results showing the higher sensitivity and specificity of the RTLS than of the EMR compared with self-reported contacts.

Table 5 shows the overall performance of combining RTLS-based and EMR-based contact tracing. If we used an “or” strategy and considered staff-patient contacts to have truly occurred if they were traced by either RTLS or EMR review, the sensitivity increased to 28/36 (77.8%); however, this came at a cost of lower specificity (179/244, 73.4%). On the other hand, if we used an “and” strategy and considered staff-patient contacts to have truly occurred if they were traced by both RTLS and EMR, the sensitivity decreased to 15/36 (41.7%) while the specificity increased to 225/244 (92.2%).

**Table 4:** Coefficients and comparisons of three logistic regression models for the EMR method, the RTLS method, and both methods.

Comparison	Model 1^a^	Model 2^b^	Model 3^c^
EMR^d^ detection, coefficient (95% CI)	1.15 (0.43 to 1.87)	N/A^e^	–0.10 (–1.01 to 0.82)
RTLS^f^ detection, coefficient (95% CI)	N/A	2.92 (2.40 to 3.74)	2.96 (2.04 to 3.88)
*P* value of nested model comparison^g^ with Model 3	.001	.84	N/A

^a^Model 1: EMR detection is the only independent variable.

^b^Model 2: RTLS detection is the only independent variable.

^c^Model 3: both EMR detection and RTLS detection are independent variables.

^d^EMR: electronic medical record.

^e^Not applicable.

^f^RTLS: real-time locating system.

^g^Compared by likelihood ratio test.

**Table 5:** Comparison of two different approaches to combining EMR and RTLS data versus self-reported contacts with patients with COVID-19. Percentages are calculated according to row values.

Self-reported status	Contact tracing using an “or” strategy		Contact tracing using an “and” strategy		Total
	Detected by EMR^a^ or RTLS^b^	Not detected by EMR or RTLS	Detected by EMR and RTLS	Not detected by EMR and RTLS	
Contacts by self-report	28 (77.8%)	8 (22.2%)	15 (41.7%)	21 (58.3%)	36
Noncontacts by self-report	65 (26.6%)	179 (73.4%)	19 (7.8%)	225 (92.2%)	244
Total	93	187	34	246	280

^a^EMR: electronic medical record.

^b^RTLS: real-time locating system.

## Discussion

### Principal Findings

Timely and accurate hospital contact tracing is vital to preserve staff and patient safety and to prevent nosocomial transmission of infectious diseases. Our study demonstrated that RTLS-based contact tracing has higher sensitivity and specificity than EMR-based contact tracing compared to self-reporting by staff.

The performance of the EMR reviews was surprisingly poor, with low sensitivity of 47% and moderate specificity of <78%. This is likely related to clinical operational processes, where the staff member performing clinical documentation for the patient may not be the same staff member who entered the patient’s room. This is especially likely in the context of a busy airborne infection isolation ward, where staff may split the workload of performing practical procedures (which would require the donning and doffing of PPE and time interacting with patients to perform clinical procedures) and administrative tasks (including clinical documentation). A lack of clarity was likely present when documenting which specific staff members had performed certain tasks. Moreover, staff-patient contacts that are not required to be noted in routine reports or that did not significantly affect clinical care of the patient may not have been documented.

Consequently, although EMR review detected more staff-patient contacts than the RTLS over our 2-day study period, many of the contacts would have either been false positive or false negative. The inability to accurately contact trace staff may lead to false alarms or false reassurances when evaluating the risk of exposure to an infectious patient.

The RTLS-based contact tracing method performed better than the EMR reviews, with moderate sensitivity of 72% and high specificity of 88%. While we did not specifically measure the time taken for each method, it was estimated that the RTLS data extraction time was approximately 2 to 3 minutes per patient, while the EMR record reviews required approximately 20 minutes per patient. These findings are comparable to previous studies validating the accuracy and ease of RFID technology in quantifying human contact episodes [12,18,19]. Chang et al [19] validated the accuracy of RFID tag readers (>80%) in detecting proximity events in the intensive care unit by comparing data obtained from direct observation; they demonstrated sensitivities ranging from 73.8%-90.9% as well as specificities ranging from 83.8%-98.0% for the technology used, with better performance for invasive events. Lucet et al [18] found no difference in the interaction duration between health care workers and patients with tuberculosis in airborne isolation when comparing records obtained from RFID network sensors, direct observations, and interviews. Hellmich et al [12] showed that the use of RFID technology in the emergency department generated twice as many contacts compared with the conventional method of EMR review during a pertussis outbreak, and each RTLS data query required less than 5 minutes, compared to 30-60 minutes per EMR review.

To identify the number of staff members in contact with an infectious patient as accurately as possible, high test sensitivity is desired. Based on our study, RTLS-based contact tracing would be able to identify most contacts with exposure to a patient with COVID-19. The sensitivity of this method could be increased slightly by integrating EMR-based contact tracing methods (with a slight compromise of specificity). Presently, this may be the most practicable solution to obtain contacts quickly. Further verifications with staff would be required to capture the most accurate list of staff contacts requiring follow-up and intervention.

Aside from staff-patient contacts, there is also potential to use RTLS technology to determine staff-staff contacts by using the RTLS data to analyze which staff members were in the same predemarcated zones at the same time. This has important practical applications in the event that a health care worker is confirmed to have an infectious disease and contact tracing of exposed staff is required.

The results obtained in our study were partially limited by implementation-related challenges of the RTLS system. RTLS electronic tags held by each staff member would need to be detected by the correct location exciter when the staff member moved from one demarcated zone to another. Careful calibration of the technology by the developers was necessary to optimize the contact tracing results.

We also observed that it was necessary to exclude a sizeable proportion of staff from our study due to staff tag–related issues. This is partly because in the setting of the COVID-19 outbreak, some nursing staff were rapidly redeployed to augment the personnel at the NCID; consequently, RTLS tags were not readily available to them. Furthermore, issues related to staff acceptance of RTLS technology, compliance with carrying the tag consistently during routine work, and technical issues such as proper tag association and regular charging of the battery may have been present. Key principles for gaining user acceptance of such technology include clearly conveying the purpose and intended uses of the technology and ensuring that individual electronic tags are practically convenient to use [13]. Although effort was made to ensure that staff are aware of the importance of contact tracing and to integrate door access into a single tag for convenient use, some staff members still did not have fully charged working tags. Further studies are needed to evaluate the knowledge, attitudes, practices, and behaviors of health care workers toward RTLS technology.

While direct observation and self-reporting methods are regarded as having a “higher” standard to accurately determine the duration of contact between staff and patients as well as the nature of the interaction (eg, multiple brief episodes versus a few episodes of prolonged contact) [6,8], such methods are not practically implementable for extended periods of time due to heavy personnel and time requirements (eg, the need for research staff to observe health care workers or for health care workers to complete self-reported diaries for an entire shift of 8-10 hours per day [6]). Other forms of technology, such as closed circuit television monitoring, have also been used to assess risk exposure of movements of health care workers in an outbreak setting [20,21]; however, these technologies are potentially time-intensive and labor-intensive.

Studies validating the accuracy of RFID technology have found no difference in the interaction duration between health care workers and tuberculosis patients when comparing records obtained from RFID network sensors, direct observations, and interviews [18] and in the detection of proximity events in the intensive care unit by comparing data from direct observation [19]. We believe that the RTLS system, when properly implemented, will be a pragmatic means of capturing all staff-patient contact episodes in a similar fashion to facilitate rapid contact management of health care workers.

### Strengths

This was a pragmatic study conducted in an outbreak setting; hence, it offers a “real-world” perspective on the usefulness of EMR-based and RTLS-based contact tracing methods. The study team employed a systematic and standardized process to perform the contact tracing using the two methods and to conduct telephone conversations with health care workers to ensure accurate capture of data. Furthermore, the findings of our study add to the limited information on the comparative effectiveness of RFID technology and conventional methods for contact tracing. Most studies on RTLSs are confined to the measurement of human contact duration in health care settings [5,22,23].

### Limitations

For the RTLS-based contact tracing, we only used location-based tracking to determine contacts and did not assess the proximity of the contacts. However, we reason that staff members entering the isolation room would likely have needed to be in close contact with the patient (within 2 meters) to perform their clinical duties; hence, this was a reasonable means of determining close contacts with the patient. Regarding our chosen standard of self-reported contacts by health care workers, information bias was possible, as staff were required to recall their movements during a shift that had already been completed. We sought to minimize errors in recall by contacting the participants 1 day after their working shifts and by corroborating their reports with other colleagues on the team. Our study period was short due to resource challenges faced by the study team, whose members were concurrently involved in outbreak management work during the COVID-19 pandemic. However, we believe that our study methodology was robust and that our observations remain valid in our evaluation of novel technology under actual outbreak conditions.

### Conclusions

We have demonstrated that RTLS-based contact tracing has higher sensitivity and specificity than EMR-based contact tracing compared with self-reported contacts. Integration of both methods appeared to provide the best performance for rapid contact tracing during the COVID-19 pandemic, with a sensitivity of 78% and a specificity of 73%. Technical adjustments and increasing user compliance are necessary to further improve the effectiveness of the RTLS for contact tracing purposes.

## Abbreviations

COVID-19: coronavirus disease
EMR: electronic medical record
NCID: National Centre for Infectious Diseases
RFID: radio-frequency identification device
RTLS: real-time locating system
